# Application of digital technology in medical practice

**DOI:** 10.3389/fpubh.2026.1722648

**Published:** 2026-02-12

**Authors:** Lihua Gu, Lu Chen, Congsha Ma

**Affiliations:** School of Nursing and Health, Shanghai Zhongqiao Vocational and Technical University, Shanghai, China

**Keywords:** digital technology, disease treatment, equipment management, medical education, medical practice

## 1 Introduction

Digital technology application in medical practice has become a key focus of stakeholders, with its rapid integration into primary care-encompassing electronic health records (EHRs), teleconsultation, home health monitoring, and more-transforming healthcare delivery (1). Supported by policies like China's 2018 Opinions on Promoting “Internet +Medical Health” (which guides trusted network construction, digital medical education, and intelligent medical device development), recent advancements (e.g., generative AI, blockchain) have further expanded its use into disease diagnosis, treatment, and service provision, unlocking great potential for improving efficiency, timeliness, and precision in public health. However, growing reliance on digital systems also raises critical challenges (data privacy, technical reliability, equitable access) that require rigorous assessment. Thus, this study critically evaluates the strengths and challenges of digital technology in medical practice, aiming to provide a nuanced perspective for its seamless, sustainable integration into primary care.

## 2 Overview of digital technology

Digital technology is a general term for the technical methods that use digitalization to collect, store, process, transmit, and present information (2). It encompasses a series of modern information technology tools and methods, including computers, the internet, big data, artificial intelligence, the Internet of Things, blockchain, and so on. Specifically, this technology has the following characteristics. First, digitization of data. Digital technology converts physical signals such as sound, images, and text into binary data that can be processed by computers, making the storage, processing, and transmission of information more flexible and efficient. Second, strong data processing capabilities. With the help of big data analysis technology, digital technology can extract valuable information from a large amount of unstructured data in a short time, assisting in decision-making and prediction (3). Third, intelligence. The development of AI technology enables digital technology not only to process data simply but also to perform pattern recognition, natural language processing, and machine learning, thus automatically completing complex tasks. Fourth, connectivity and interoperability. Digital technology can enable the connection between devices and systems through the internet and other communication means, allowing information to be seamlessly shared across different devices and platforms. Fifth, strong scalability and flexibility. The architecture of digital technology can be expanded and updated according to changes in demand. The modular design of software systems and hardware devices also makes technological upgrades and functional expansion easier. Sixth, data visualization. Digital technology can transform complex data into intuitive graphics and images, thereby helping users better understand the information.

Digital technology, which integrates an array of contemporary informational tools including big data, artificial intelligence (AI), the Internet of Things (IoT), and blockchain, presents both substantial opportunities and challenges within the domain of medicine. The deployment of such technology necessitates careful consideration of several critical issues. Foremost among these is the potential for data processing bias, wherein the efficacy of big data and AI systems is contingent upon the caliber and variety of the training datasets employed. An absence of diversity within these datasets may lead to biased predictions or diagnostic inaccuracies, with a disproportionate effect on underrepresented populations. Furthermore, there is a palpable concern regarding an over dependence on technological solutions, as there exists a risk that healthcare professionals may assign excessive confidence to AI and automated systems. Such reliance could potentially erode the indispensable human elements of clinical judgment and empathy that are integral to the provision of patient care.

## 3 Specific applications of digital technology in medical practice

### 3.1 Application in the management of medical institutions

Digital technology has transformed hospital management by automating personnel scheduling, financial processes, and equipment monitoring. Despite these benefits, critical issues persist:

#### 3.1.1 Personnel management

In terms of personnel management, digital technology is mainly used for the development of intelligent scheduling systems, Human Resource Management Systems (HRMS), and performance management systems. Among these, the intelligent scheduling system utilizes algorithms to automatically generate scheduling plans based on personnel skills, availability, and hospital demand, and can be dynamically adjusted in real-time to ensure the rational allocation of medical and nursing resources and improve work efficiency (4). Research data indicate that the incidence of depression among shift nurses reaches 58.82%, with anxiety rates as high as 62.08%. These emotional issues are closely associated with multiple factors, including fatigue during shifts, psychological stress before and after night shifts, post-rest recovery, medication use for sleep during night shifts, physical discomfort during shifts, workload intensity, dietary habits during shifts, weekly work hours exceeding 40 hours, and sleep quality before and after night shifts (5). To address this situation, various intelligent algorithms have been applied to optimize nurse scheduling, significantly improving satisfaction among hospital nurses, their families, and patients. Through multi-objective programming models, these algorithms effectively balance conflicts between sub-objectives, enhancing overall scheduling efficiency. This approach ensures equitable distribution of working hours for each nurse while preventing overexertion in certain shifts (6). The HRMS integrates processes such as recruitment, onboarding training, attendance, and compensation management through digital technology, achieving automation and standardization of internal personnel management in medical institutions and reducing the error rate of manual operations. The performance management system, by recording employees' work performance and training progress, provides data support for the development of personalized training plans and performance evaluations, thereby enhancing the overall quality of the medical team. Nevertheless, intelligent scheduling systems may inadvertently disregard human factors, such as team dynamics or personal preferences, which could result in discontent among the personnel.

#### 3.1.2 Financial management

In financial management, digital technology is mainly used in Financial Information Systems (FIS), electronic invoice systems, and budget management platforms, thereby optimizing the financial processes of medical institutions. Among these, the financial information system is capable of integrating and automatically processing various financial data of medical institutions, including income, expenses, cost accounting, and report generation, improving the accuracy and efficiency of financial data processing (7). The electronic invoice system simplifies the charging and reimbursement processes and reduces the burden of manual operations through automation, lowering the risk of errors and omissions in accounting. The budget management platform utilizes digital technology and big data analysis techniques to predict future trends in income and expenses, assisting the finance department in formulating reasonable budget plans, achieving efficient resource allocation, and effective cost control. However, the dependence on financial management platforms may expose vulnerabilities to cyber-attacks, thereby potentially jeopardizing the integrity of sensitive financial information.

#### 3.1.3 Materials and equipment management

In the management of materials and equipment, digital technology is mainly applied in medical equipment management systems, material supply chain management systems, and asset management platforms. Among these, the medical equipment management system can collect real-time operational data through sensors installed on the equipment and aggregate this data using digital technology and the Internet of Things. It then utilizes big data analysis to predict fault trends, thereby improving the management of materials and equipment, reducing downtime, and extending the lifespan of the equipment (8). A controlled study demonstrated that the implementation of the PDCA cycle (Plan-Do-Check-Act) management tool for medical devices reduced the failure rate from 2.5 times per 1,000 h to 1.2 times per 1,000 h, while the average repair time after a failure decreased from 8 ± 0.7 h to 5 ± 0.5 h (9). This significantly improved equipment operation and maintenance efficiency as well as clinical support capabilities. This not only ensures the continuity of clinical services but also effectively extends equipment lifespan by avoiding costly emergency repairs and maximizing utilization, thereby delivering substantial return on investment. The material supply chain management system achieves automated management of procurement, inventory, and distribution of materials through a digital platform, thereby optimizing the supply chain process and ensuring the timely supply of medical materials. The asset management platform records the usage, location, and maintenance history of equipment and materials, thereby improving the utilization rate of materials, avoiding duplicate purchases, and preventing resource wastage (10). However, predictive maintenance systems are contingent upon Internet of Things (IoT) devices, which could become vectors of failure in the event that cybersecurity measures are insufficiently robust.

### 3.2 Application in disease diagnosis and treatment

In the practice of disease diagnosis and treatment, digital technology is mainly used to acquire and provide relevant information support, enabling medical staff to carry out medical activities more accurately and effectively.

#### 3.2.1 Disease diagnosis

In disease diagnosis, digital technology is mainly used in AI-assisted diagnosis, Clinical Decision Support Systems (CDSS), and other aspects. Among these, AI-assisted diagnosis systems use deep learning algorithms to automatically analyze medical images (such as *X*-rays, CT, and MRI) to detect abnormal lesions, assisting doctors in early diagnosis of diseases such as cancer and stroke, and improving the speed and accuracy of diagnosis (11). Clinical Decision Support Systems integrate patients' medical history, genomic data, laboratory test results, and medical imaging information to provide doctors with personalized diagnostic recommendations and treatment plans, reducing the incidence of misdiagnosis and missed diagnosis (12). Al-assisted diagnosis has shown promise in improving speed and accuracy. However, algorithms trained on limited datasets risk being less effective for populations not represented in the data. Additionally over-reliance on Al might lead to complacency in human oversight, increasing the likelihood of errors in complex cases.

#### 3.2.2 Disease treatment

In disease treatment, digital technology has promoted the widespread application of surgical navigation systems, personalized treatment, intelligent nursing systems, smart beds, and nursing robots. Among these, surgical navigation systems, combining virtual reality (VR), augmented reality (AR), and robotic technology, assist surgeons in performing high-precision minimally invasive surgeries, improving the safety and effectiveness of operations through real-time image guidance (13). Personalized treatment platforms use patients' genetic information, medical history, and lifestyle data to formulate targeted treatment plans, significantly improving treatment outcomes, especially in cancer and chronic disease management. Intelligent nursing systems can monitor patients' vital signs (such as heart rate, blood pressure, oxygen saturation, etc.), providing timely alerts for necessary interventions. Smart beds and nursing robots assist nursing staff in completing routine patient care tasks, such as adjusting body positions, administering medication, and arranging meals, reducing the workload of nursing staff (14). Additionally, digital technology records patients' nursing plans, implementation, and assessments through Nursing Information Systems (NIS), promoting the standardization and personalization of nursing work and ensuring that patients receive comprehensive and continuous care services (15). While surgical navigation systems and personalized treatments offer significant advancements, their success depends on the reliability of underlying technology. Failures in these systems could lead to adverse patient outcomes. Furthermore, ethical concerns arise when Al-driven systems suggest treatments without clear explain ability.

### 3.3 Application in medical skills training

Digital technology has provided various new methods for the conduct of medical skills training activities, significantly improving the quality and effectiveness of training for doctors and nurses. Specifically, the application of this technology in this area is mainly manifested in the following aspects.

#### 3.3.1 Virtual reality (VR) and augmented reality (AR) technologies

VR and AR technologies have created an immersive learning experience environment for medical skills training, allowing medical staff to practice operations in a simulated environment. For example, with the help of VR technology, surgeons can simulate complex surgical scenarios and anatomical structures, practice surgical procedures in a risk-free virtual environment, and repeatedly practice specific steps to enhance the precision of surgery (16). A cross-over controlled study published in 2024 involving three mainstream virtual reality simulators demonstrated that VR-trained trainees exhibited significant improvements in laparoscopic basic skill tasks, with a 44% reduction in median instrument manipulation path length and a 45% decrease in median task completion time, indicating substantial progress in both operational efficiency and precision (17). Moreover, AR technology can overlay digital anatomical information onto actual operations, providing real-time guidance for doctors performing minimally invasive surgery or needle puncture procedures. In nursing skills training, VR technology can be used for training in emergency operation skills such as simulated cardiopulmonary resuscitation (CPR) or trauma care, helping nurses practice correct methods and steps in emergency situations, improving their emergency response capabilities and reaction speed.

#### 3.3.2 Simulation training

Digital technology can be used to support the development and utilization of simulation training systems, thereby providing doctors and nurses with highly realistic operation training platforms, allowing them to engage in training ranging from basic operations to complex surgeries. For example, doctors can use simulation training systems to practice high-difficulty operations such as heart surgery, minimally invasive interventional surgery, and endotracheal intubation. Moreover, the system provides real-time feedback on the precision of the operations and changes in the patient's vital signs, helping to improve their operational skills and strategies. Nurses can use the system's simulated dolls and virtual ward training functions to practice basic nursing skills such as intravenous infusion, wound care, and catheterization. The system also provides real-time feedback on the correctness of the operations, ensuring the precision of nursing skills.

#### 3.3.3 Online learning platforms

The rapid development of digital technology has broken through the traditional limitations of time and space in medical education and training, allowing doctors and nurses to engage in personalized learning through online learning platforms such as WeChat mini-programs, adapting to the increasingly complex medical needs (18). Online learning platforms not only provide a wealth of learning resources for medical staff but also create a flexible learning environment that can meet the learning needs of medical staff at different stages. For doctors, online platforms offer a variety of learning resources, including surgical video demonstrations, clinical case discussions, diagnostic and treatment guidelines, and academic lectures. Through surgical video demonstrations, one can gain a detailed understanding of the steps and techniques of various surgeries, especially those that are more complex or emerging, such as minimally invasive surgery and robot-assisted surgery. In addition, the clinical case discussion module offers different types of disease treatment plans and best practices, which doctors can reference to enhance their clinical decision-making abilities. Moreover, online platforms can recommend courses based on their professional fields (such as internal medicine, surgery, obstetrics and gynecology, etc.), supporting specialized further education. At the same time, they can also access the latest diagnostic and treatment guidelines and clinical research through online platforms, keeping pace with the forefront of medical development and continuously updating their professional knowledge and skills. For nurses, online learning platforms also offer a wealth of learning resources covering basic nursing, emergency skills, medication management, and patient communication skills. The platform features simulated nursing scenarios and operational videos, which can help nurses master the essentials of basic skills and complex nursing procedures, improving the accuracy and standardization of actual operations. Online courses on basic nursing skills, specialized nursing knowledge, and intensive care techniques meet the training needs of different nursing professionals. Furthermore, online learning platforms not only provide self-study courses but also support interactive learning. Medical staff can communicate with peers and experts through online seminars, real-time Q&A, and virtual discussion areas to broaden their horizons, increase their understanding of complex issues, and also enhance the collaboration and collective wisdom of medical teams.

#### 3.3.4 Remote skills training

The application of digital technology in remote skills training allows medical staff in remote areas to participate in real-time surgical demonstrations, skill practice exercises, and case discussions through video conferencing, online live streaming, and remote simulation training, thus breaking through the limitations of geography and time on medical skills training (19). Additionally, remote training can simulate the handling process of emergency events, helping medical staff better cope with various urgent situations in their actual work. Moreover, remote skills training can also be used for skill exchange and collaborative training between multiple institutions. For example, several hospitals or medical institutions can jointly conduct training activities through a remote training platform, discussing complex surgical cases or treatment plans for difficult diseases. Through this form of joint training, medical staff from different institutions can share each other's experiences and technical advantages, enhancing the overall level of medical skills.

### 3.4 Application in out-of-hospital medical services

In addition to a wide range of applications in in-hospital medical practice, digital technology also has many applications in out-of-hospital medical services, especially with the development of digital medical services in recent years. Overall, the applications in this area can be summarized as follows.

#### 3.4.1 Telemedicine and online consultations

With the support of digital technology, medical institutions can use telemedicine platforms to provide patients with relevant medical services. Patients can interact with doctors through video consultations, voice calls, and instant messaging, which is particularly suitable for the elderly who have difficulty moving or patients living in remote areas (20). Telemedicine can significantly improve healthcare accessibility for residents in rural areas. In low-and middle-income countries, the implementation of telemedicine effectively reduces the need for patients to travel long distances to medical facilities, saving time and transportation costs associated with medical visits. Studies indicate that telemedicine can avoid the economic burden of patients traveling long distances to obtain specialized medical services (21). Although the specific savings vary by region and service type, its potential to reduce healthcare costs has been widely recognized. Moreover, these platforms enable doctors to conduct disease diagnosis, medication guidance, and rehabilitation advice, thereby reducing the burden of travel for patients. For some special cases or emergencies, doctors can also conduct preliminary assessments through telemedicine and decide whether further offline treatment is needed.

#### 3.4.2 Smart drug delivery and home medication management

The application of digital technology in out-of-hospital medication management has promoted the intelligent development of drug ordering, delivery, and management. Patients can order prescription or over-the-counter medications through online platforms, and the drugs will be delivered directly to their homes by pharmacies, reducing the hassle of going to the hospital or pharmacy to purchase medications. This is particularly beneficial for chronic patients who require long-term medication and the elderly who have difficulty moving.

#### 3.4.3 Mobile health applications (mHealth)

Currently, a variety of health management apps and wearable devices available in the market support daily health management and disease prevention by recording users' health data, such as blood pressure, blood sugar, steps, sleep quality, etc. For patients with chronic diseases, these applications can provide visual feedback on condition tracking, symptom recording, and treatment progress, helping patients and doctors better understand the changes in the condition and optimize treatment plans (22). Additionally, mobile health applications can also offer personalized health advice, diet plans, and fitness guidance, encouraging patients to manage their health and improve their lifestyle at home.

#### 3.4.4 Digital support for home rehabilitation and nursing

For patients in the postoperative recovery period or elderly individuals requiring long-term care, digital technology offers remote rehabilitation guidance and support from intelligent nursing devices. For instance, home rehabilitation platforms developed by medical institutions can provide patients with guided videos for rehabilitation training and remote assessments, allowing them to complete daily rehabilitation exercises at home. Meanwhile, doctors or therapists can also adjust the rehabilitation plan by monitoring and feedback data through video (23).

#### 3.4.5 Smart integrated medical and elderly care service model

The application of digital technology in the smart integrated medical and elderly care service model has promoted the deep integration of medical and elderly care resources, providing convenient and efficient health services for patients and the elderly. For example, with the support of digital technology, a full-chain service model can be constructed through the Internet of Things, Artificial Intelligence (AI), and big data platforms, integrating data from hospitals, communities, and households to achieve the full lifecycle management of patients' electronic health records (24). Wearable devices are used for real-time monitoring of patients' vital signs and uploading the data to the medical platform, assisting doctors and caregivers in precise interventions and reducing the risk of sudden incidents. By coordinating medical institutions, nursing homes, communities, and households through digital technology, a multi-subject integrated service model is formed, with the smart elderly care platform enabling data sharing and service collaboration among multiple subjects. Community nurses, family doctors, and hospital doctors can access patients' health information through the platform to jointly develop personalized care plans. Patients can receive consultations and guidance using remote medical equipment at home, with community centers promptly tracking their recovery and adjusting services based on actual needs. Additionally, the “proactive health” model of traditional Chinese medicine, combined with digital technology, provides health management for the elderly (25). Smart wearable devices monitor patients' vital signs such as pulse and blood pressure, and combined with TCM data analysis, offer personalized health advice, such as massage, dietary, and exercise guidance. The elderly can obtain personalized TCM health conditioning plans through a health management app, achieving disease prevention and chronic disease management.

## 4 Challenges and limitations

### 4.1 Data security and privacy protection

The widespread application of digital technology in medical practice has made data security and privacy protection issues increasingly prominent. For example, digital medical tools such as electronic medical record systems, telemedicine platforms, and health management applications store a large amount of user information, especially highly sensitive content such as patients' medical history, genetic information, and diagnostic records, which can easily become targets for cyber attacks. Therefore, it is essential to provide security protection for these data. Firstly, advanced encryption technologies such as end-to-end encryption and Transport Layer Security (TLS) protocols should be adopted to protect data security and prevent information from being intercepted or tampered with during transmission. Secondly, biometric recognition technologies (such as fingerprint recognition, facial recognition) should be combined for security reinforcement to ensure that only authorized personnel can access sensitive data, thus enhancing the overall protection level of the system. Thirdly, regular cybersecurity audits and system penetration tests should be conducted, especially for the key nodes and storage locations of medical systems, to discover and repair potential security vulnerabilities in a timely manner. Finally, with the widespread use of remote monitoring devices, smart beds, and wearable devices in medical practice, IoT devices may become a weak link for cyber attacks. Therefore, it is necessary to establish comprehensive security management strategies, including strict security checks and continuous risk assessments, to ensure the safety of digital technology applications.

### 4.2 Technical reliability and system stability

The application of digital technology raises higher requirements for the reliability and stability of related systems, especially the operation of surgical navigation systems and telemedicine platforms, which must possess a high degree of stability and reliability. Therefore, it is necessary to design redundancy mechanisms in related medical systems and platforms to prevent single points of failure from affecting the entire system. At the same time, routine maintenance and regular updates should be conducted on related equipment and software. In this regard, medical institutions should develop detailed equipment maintenance plans, including regular hardware inspections, system calibration, and timely software upgrades and security patch installations, to prevent medical risks caused by technical failures. Additionally, distributed storage and cloud storage tools should be used for multi-level data backups to ensure that data can be quickly recovered and services can continue in the event of system crashes or large-scale failures, thus avoiding impacts on patient health.

### 4.3 Limitations of artificial intelligence and algorithmic bias

The application of digital technology in medical practice relies on algorithm training with a large amount of data. If the data used for training is biased or unbalanced, it may lead to poor performance of the algorithm. For example, if the training data primarily comes from a specific population, artificial intelligence may not be able to make accurate judgments when faced with other populations. Therefore, it is necessary to ensure the diversity and representativeness of the dataset. At the same time, the application scope of artificial intelligence technology should be strictly defined to avoid directly relying on artificial intelligence to make diagnostic or treatment decisions in complex cases with insufficient data support or multiple variables. Doctors need to make comprehensive judgments by combining the patient's medical history, clinical symptoms, and the analysis results of artificial intelligence to ensure the accuracy of diagnosis and the safety of treatment.

Al algorithms trained on unrepresentative datasets may exacerbate health care disparities. Furthermore, the opacity of many Al systems raises ethical concerns regarding accountability and patient trust. Transparent algorithms and inclusive datasets are necessary to address these issues.

## 5 Conclusion

The application of digital technology in medical practice is increasingly widespread and has had a profound impact on the efficiency, quality, and precision of medical services. This paper systematically reviews the specific applications of digital technology in medical institution management, disease diagnosis and treatment, equipment management, and medical education, demonstrating its important role in improving the efficiency of medical management, optimizing diagnostic processes, enhancing treatment precision, and advancing medical education. At the same time, the application of digital technology also faces a series of challenges, including data security and privacy protection, system reliability and stability, limitations and bias issues of artificial intelligence algorithms, which require comprehensive management through technical means and institutional guarantees. It can be seen that digital technology brings many advantages to medical practice, but its effective implementation needs to balance technological innovation with potential risks and strengthen technical management and compliance to achieve sustainable improvement and development of medical services.

The integration of digital technology in medical practice has undoubtedly brought transformative benefits, improving efficiency, precision, and accessibility. However, these advancements must be critically examined to ensure equitable and ethical application. Challenges such as data security system reliability, and algorithmic bias require proactive management. By balancing innovation with critical oversight, the healthcare sector can harness the full potential of digital technology while safeguarding against its risks.
